# *FGFR2* gene polymorphism rs2981582 is associated with non-functioning pituitary adenomas in Chinese Han population: a case–control study

**DOI:** 10.1042/BSR20181081

**Published:** 2018-11-09

**Authors:** Bin Zhu, Juan Wang, Lingling Qin, Lei Wang, Yanfei Zheng, Lei Zhang, Wei Wang

**Affiliations:** 1 School of Traditional Chinese Medicine, Beijing University of Chinese Medicine, Beijing 100029, China; 2 Department of Pharmacy, Beijing Tiantan Hospital, Capital Medical University, Beijing 100050, China; 3 Education Research Evaluation Center, Beijing University of Chinese Medicine, Beijing 100029, China; 4 Technology Department, Beijing University of Chinese Medicine, Beijing 100029, China; 5 Department of Endocrinology, Third Affiliated Hospital of Beijing University of Chinese Medicine, Beijing 100029, China; 6 Department of Pharmacy, Beijing Shijitan Hospital, Capital Medical University, Beijing 100050, China

**Keywords:** FGFR2, Genotype, Non-functioning pituitary adenoma, Polymorphism

## Abstract

The association of the fibroblast growth factor receptor 2 gene (*FGFR2*) polymorphism rs2981582 with breast cancer has been extensively studied, whereas the role of this polymorphism in non-functioning pituitary adenoma (NFPA) has not been elucidated. We thus investigated a potential association of rs2981582 with NFPA. A total of 79 patients and 142 healthy control participants were enrolled in our study. DNA of the participants was extracted from peripheral blood samples and genotyped by using the MassARRAY method. We found that the AA genotype was associated with a higher risk of developing NFPA (OR = 1.743, 95%CI: 1.151–2.64, *P*=0.008). After adjusting for risk factors, significant difference was still observed between the two groups (OR = 1.862, 95%CI: 1.172–2.957, *P*=0.008). Moreover, under the assumptions of the recessive model (OR = 3.051, 95%CI: 1.403–6.635, *P*=0.005) and the additive model (AG: OR = 0.329, 95%CI: 0.144–0.755, *P*=0.009; AA: OR = 0.326, 95%CI: 0.141–0.757, *P*=0.009), rs2981582 was associated with an increased risk of NFPA. Our results proved that *FGFR2* rs2981582 AA genotype was associated with a higher risk of NFPA. The recessive model and additive model also showed increased the risk of NFPA.

## Introduction

Non-functioning pituitary adenomas (NFPAs) are benign pituitary neoplasms that arise from adenohypophyseal cells and are not associated with any clinical syndrome related to hormone overproduction from the tumor [1,2]. NFPAs constitute 15–37% of all pituitary adenomas and have a prevalence of 70–220 per million in the population [3]. It is the most common type of adult pituitary macroadenoma, and its most frequent clinical symptoms are headache, visual defects, and hypopituitarism [4–6]. As the clinical manifestations of hormonal hypersecretion are absent, the diagnosis of such patients is always delayed [7].

Several studies have explored the relationships between single nucleotide polymorphism (SNP) sites and pituitary adenoma (PA) in general by large genome-wide association studies (GWAS) [8–10]. However, no studies examined the relationship of SNPs with NFPA. Fibroblast growth factor receptor 2 (FGFR2) is a member of the FGFR family of tyrosine kinase receptors. The *FGFR2* gene is located on chromosome 10q26 and contains 22 exons [11,12]. FGFR2 participates in the process of tumorigenesis by mediating mitogenic and survival signals as well as by promoting invasiveness and angiogenesis [13]. Variants of the *FGFR2* polymorphism rs2981582, which was first identified by Easton et al. in a large-scale GWAS, were most commonly reported to be associated with breast cancer or prostate cancer [14,15]. Glebauskiene et al. observed that the *FGFR2* rs2981582 G/G genotype was observed less frequently in the non-invasive PA subgroup than in healthy controls, whereas the G/A genotype was more frequently observed in the non-invasive PA subgroup than either in control group or invasive PA subgroup [16]. Nevertheless, given that the relationship between rs2981582 variants and specifically NFPA is still unknown, we sought to assess the impact of *FGFR2* rs2981582 on sporadic NFPA and determine a possible association of rs2981582 variants with NFPA risk in Chinese population in a case–control study.

## Materials and methods

### Patient recruitment

Participants were consecutively recruited from June to December in 2017 among inpatients in the Beijing Tiantan Hospital. The inclusion criteria were as follows: (1) diagnosis of PA in patients confirmed via magnetic resonance imaging (MRI); (2) normal laboratory tests for the growth hormones prolactin and adrenocorticotropic hormone; (3) absence of other serious diseases. In the control group, age, gender, symptoms, physical examinations, and medical history were recorded. Control group participants were enrolled among healthy individuals after examination that did not reveal any disease or family history of related diseases. Patients with severe hepatic, renal, or cardiac impairment, or any serious illness were excluded. The study was performed according to the guidelines of the Declaration of Helsinki, and was approved by the Ethics Committee of the Beijing Tiantan Hospital. A signed informed consent was obtained from all patients or from their direct relatives.

### Genotyping

Blood samples of each participant were collected into tubes containing ethylenediaminetetraacetic acid and stored at −80°C until use. Genomic DNA was extracted from the whole blood using a commercial kit (Qiagen, Hilden, Germany) according to the manufacturer’s protocol. DNA quantity was examined by a NanoDrop2000 spectrophotometer (Thermo Fisher, Waltham, MA, U.S.A.). Polymerase chain reaction (PCR) primer pairs used to amplify *FGFR2* rs2981582 were as follows: ACTGCTGCGGGTTCCTAAAG (forward); CACTCATCGCCACTTAATG (reverse). Genotyping was performed by using time-of-flight mass spectrometry on a MassARRAY iPLEX platform (Sequenom, San Diego, CA, U.S.A.) by Bio Miao Biological Technology (Beijing, China). The average genotype call rate for this SNP was >98%.

### Statistical analysis

All statistical analyses were performed by SPSS (version 17.0, SPSS Inc., Chicago, U.S.A.). The Hardy–Weinberg equilibrium (HWE) was assessed by using a χ^2^ test and a *P*-value <0.05 was considered to indicate significant disequilibrium. Differences in genotypic frequencies between groups were calculated by the Pearson’s χ^2^ test or Mann–Whitney *U* test. Odds ratio (OR) values with 95%CI were calculated. Logistic regression analysis was used to evaluate the contribution of genetic and non-genetic factors to NFAP risk. Patients’ gender, age, bodyweight, smoking, and drinking habits were treated as risk factors to be adjusted in the analysis.

## Results

### Characteristics of the study participants

A total of 79 patients (38 female) with a mean age of 41.37 ± 11.63 years and bodyweight 72.26 ± 13 kg were enrolled in this hospital-based case–control study. All patients were Chinese and with confirmed diagnosed of NFPA. Of them, 24.5% had a history of smoking and 13.9% were drinking alcohol. The control group enrolled 142 healthy persons (73 female) with a mean age of 42.12 ± 11.63 years and bodyweight 69.04 ± 14.35 kg. Nearly 17.6% and 16.8% of control group participants smoked and consumed alcoholic beverage, respectively. No deviation from the HWE was observed (HWE = 0.236, *P*>0.05). The minor allele frequency was 0.3455. Demographic characteristics of the participants are given in Table 1.

**Table 1:** The basic characteristic of the enrolled patients

	Control	Case
Gender (M/F)	142 (69/73)	79 (41/38)
Age	42.12 ± 11.63	41.37 ± 11.63
Bodyweight	69.04 ± 14.35	72.26 ± 13
Smoking (%)		
Yes	17.6%	24.5%
No	82.4%	75.9%
Drinking (%)		
Yes	16.8%	13.9%
No	83.2%	86.1%

### Genetic association analysis

Genotype and allele frequencies of the *FGFR2* gene polymorphisms in patients and healthy controls are shown in Table 2. Significant differences were observed between patients and healthy controls in the distribution of AA, GA, and GG genotypes (*P*=0.018). In addition, there was also a significant difference in the recessive model (*P*=0.007). To analyze the influence of gender on the development of NFPA, we also divided all participants into males and females. There were no significant differences in the frequencies of AA, GA, and GG between patients and healthy controls in males or females considered separately. However, we found that there was a difference in the recessive model (AA vs. GA + GG) in females (Table 3).

**Table 2:** The genotype of FGFR2 rs2981582 polymorphism in case and control group

rs2981582	Control	Case	*P*
AA	13	18	0.018
GA	62	28	
GG	68	31	
A	88	64	0.027
G	198	90	
AA + AG vs. GG	75/68	46/31	0.32
AA vs. AG + GG	13/130	18/59	0.007

**Table 3:** The genotype of FGFR2 rs2981582 polymorphism with different gender

	Male			Female		
	Case	Control	*P*	Case	Control	*P*
AA	9	7	0.223	9	6	0.057
GA	16	32		12	30	
GG	16	31		15	37	
A	34	46	0.247	30	42	0.067
G	48	94		42	104	
AA + AG vs. GG	25/16	39/31	0.691	21/15	36/37	0.419
AA vs. AG + GG	9/32	7/63	0.099	9/27	6/67	0.035

The logistic regression analysis of rs2981582 SNP genotype between patients and healthy controls was also performed. We found that the AA allele significantly correlated with NFPA morbidity (OR = 1.743, 95%CI: 1.151–2.64, *P*=0.008) even after adjusting for gender, age, bodyweight, smoking, or drinking habits (OR = 1.862, 95%CI: 1.172–2.957, *P*=0.008) (Table 4). In addition, the effects of genetic models (dominant, recessive, and additive models) on NFPA risk were also observed between two groups. We did not found any significant associations of rs2981582 with NFPA in the dominant model (OR = 1.345, 95%CI: 0.7674–2.359, *P*=0.300). However, rs2981582 had a significant impact in the recessive model (OR = 3.051, 95%CI: 1.403–6.635, *P*=0.005) and the additive model (AG: OR = 0.329, 95%CI: 0.144–0.755, *P*=0.009; AA: OR = 0.326, 95%CI: 0.141–0.757, *P*=0.009), even after adjusting for gender, age, bodyweight, smoking, or drinking habits (Table 5).

**Table 4:** The logistic regression analysis of rs2981582 SNP genotype with the morbidity of NFPA

rs2981582	OR	95%CI	*P*	OR*	95%CI*	P*
GG	Reference			Reference		
AA	1.743	1.151–2.64	**0.008**	1.862	1.172–2.957	**0.008**
GA	0.5684	0.309–1.046	0.069	0.6539	0.3365–1.27	0.021

* Adjusting for gender, age, bodyweight, smoking, and drinking. Significant associations are marked in bold.

**Table 5:** The logistic regression analysis of the dominant model, recessive model, and additive model in case and control group

rs2981582	OR	95%CI	*P*	OR*	95%CI*	P*
Dominant model (GA + AA vs.GG)	1.345	0.7674–2.359	0.300	1.622	0.8784–2.995	0.122
Recessive model (AA vs. GA + GG)	3.051	1.403–6.635	**0.005**	3.153	1.329–7.481	**0.009**
Additive model						
AG	0.329	0.144–0.755	**0.009**	0.325	0.141–0.749	**0.008**
AA	0.326	0.141–0.757	**0.009**	0.310	0.133–0.722	**0.007**

* Adjusting for gender, age, bodyweight, smoking, and drinking. Significant associations are marked in bold.

## Discussion

In the absence of clinical manifestations of hormonal hypersecretion, NFPAs are usually found when they are large enough to exert pressure on surrounding structures [17]. Until now, there are no effective drugs available for NFPA, and surgical resection remains the first-line treatment [18]. However, a complete resection is achieved only in 40–50% of the cases, and at least 10–20% of completely resected tumors recur after 5–10 years [5]. As a consequence, the long-term survival of individuals with NFPA is compromised.

NFPA emergence involves a complex combination of genetic, environmental, and lifestyle factors. Inherited susceptibility makes an important contribution to cancer development [19]. Identifying and understanding the functional importance of genetic alterations is crucial to extend our understanding of NFPA. Although *FGRF2* polymorphism rs2981582 has been extensively evaluated in breast and prostate cancers, the role of this polymorphism in NFPA patients has not been elucidated yet [11,20–22]. Our results have demonstrated that *FGFR2* rs2981582 AA genotype or the recessive model was associated with a higher risk of NFPA. To the best of our knowledge, this is the first study of the relationship between *FGFR2* polymorphism rs2981582 and NFPA.

The relationship of *FGFR2* rs2981582 polymorphism with PA was also reported by Glebauskiene et al. [16]. However, there were several differences between our study and theirs. First, the enrolled patients were different. Our study only enrolled NFPA patients, whereas Glebauskiene et al. enrolled patients with PA in general. Second, neither of the three rs2981582 genotypes had statistically different prevalence in the overall PA group compared with that in controls in their Caucasian population, although in a more detailed analysis, *FGFR2* rs2981582 G/G genotype was less prevalent in non-invasive PA cases than in healthy controls [16]. In contrast, in our study in Chinese population, we found that individuals with rs2981582 AA genotype may have a higher NFPA risk. In addition, by using binomial logistic regression analysis, we showed that rs2981582 had a significant effect in the recessive and additive models.

Although our results are promising and might be useful in helping to stratify patients, they should be considered as preliminary ones and further research is necessary. There were also some limitations that may not be ignored. First, as the mechanism of NFPA is dramatically complex, and we only focused on a polymorphism in one gene, this cannot completely explain the morbidity. Furthermore, the function of FGFR2 in NFPA is still unclear, and more experimental data on this subject are required. Second, as the sample size was limited in our study, several indices bordered the level of statistical significance. In this regard, larger cohorts of patients and healthy controls will be needed in future studies. Third, although we chose the control subjects strictly from the individuals deemed to be healthy by necessary examinations, they did not undergo MRI diagnostics to fully exclude the PA risk. Thus, in future, even stricter inclusion and exclusion criteria should be adopted.

## Conclusion

In summary, we for the first time showed an association of *FGFR2* polymorphism rs2981582 with NFPA. However, this result needs to be replicated in a larger sample of patients, and functional studies will be necessary to investigate whether and how this polymorphism might affect NFPA pathogenesis.

## Abbreviations

GWAS: Genome-Wide Association Studies
HWE: Hardy–Weinberg equilibrium
NFPA: non-functioning pituitary adenoma
PA: pituitary adenoma
SNP: single nucleotide polymorphism
